# GEMmaker: process massive RNA-seq datasets on heterogeneous computational infrastructure

**DOI:** 10.1186/s12859-022-04629-7

**Published:** 2022-05-02

**Authors:** John A. Hadish, Tyler D. Biggs, Benjamin T. Shealy, M. Reed Bender, Coleman B. McKnight, Connor Wytko, Melissa C. Smith, F. Alex Feltus, Loren Honaas, Stephen P. Ficklin

**Affiliations:** 1 Molecular Plant Sciences Program, Washington State University, Pullman, WA USA; 2 Department of Horticulture, Washington State University, Pullman, WA USA; 3 Department of Electrical and Computer Engineering, Clemson University, Clemson, SC USA; 4 Biomedical Data Science and Informatics, Clemson University, Clemson, SC USA; 5 Department of Genetics and Biochemistry, Clemson University, Clemson, SC USA; 6 Department of Electrical Engineering and Computer Science, Washington State University, Pullman, WA USA; 7 Center for Human Genetics, Clemson University, Greenwood, SC USA; 8 USDA Agricultural Research Service, Wenatchee, WA USA

**Keywords:** RNA-seq, Workflows, Gene expression matrix, Gene co-expression network, Differential gene expression, Nextflow

## Abstract

**Background:**

Quantification of gene expression from RNA-seq data is a prerequisite for transcriptome analysis such as differential gene expression analysis and gene co-expression network construction. Individual RNA-seq experiments are larger and combining multiple experiments from sequence repositories can result in datasets with thousands of samples. Processing hundreds to thousands of RNA-seq data can result in challenges related to data management, access to sufficient computational resources, navigation of high-performance computing (HPC) systems, installation of required software dependencies, and reproducibility. Processing of larger and deeper RNA-seq experiments will become more common as sequencing technology matures.

**Results:**

GEMmaker, is a nf-core compliant, Nextflow workflow, that quantifies gene expression from small to massive RNA-seq datasets. GEMmaker ensures results are highly reproducible through the use of versioned containerized software that can be executed on a single workstation, institutional compute cluster, Kubernetes platform or the cloud. GEMmaker supports popular alignment and quantification tools providing results in raw and normalized formats. GEMmaker is unique in that it can scale to process thousands of local or remote stored samples without exceeding available data storage.

**Conclusions:**

Workflows that quantify gene expression are not new, and many already address issues of portability, reusability, and scale in terms of access to CPUs. GEMmaker provides these benefits and adds the ability to scale despite low data storage infrastructure. This allows users to process hundreds to thousands of RNA-seq samples even when data storage resources are limited. GEMmaker is freely available and fully documented with step-by-step setup and execution instructions.

**Supplementary Information:**

The online version contains supplementary material available at 10.1186/s12859-022-04629-7.

## Background

Transcriptome sequencing (RNA-seq) is used in the life sciences to explore gene–gene and gene-trait relationships [37]. The full workflow for an RNA-seq experiment consists of several steps including experimental design, RNA collection, cDNA library construction sequencing, read cleaning, transcript mapping and gene expression quantification. Downstream computational analyses vary depending on the research goal, and can include differential gene expression (DGE) [20, 29], gene regulatory network construction [6, 23], eQTL analysis [32, 41], and gene co-expression network (GCN) analysis [18, 30].

Individual RNA-seq experiment increasingly include hundreds to thousands of samples. These experiments are often made available on public repositories–such as the National Center for Biotechnology Information (NCBI) [25]–allowing them to be mined for new knowledge. To prepare RNA-seq data for downstream computational analysis, expression levels must first be quantified, which is the process of converting raw RNA-seq reads to count data. Count data is stored as a gene expression matrix (GEM) which is an *n* x *m* matrix of *n* genes and *m* samples with values representing gene expression levels. Quantification of gene expression levels is performed using popular tools such as HISAT2 [14], Salmon [26], kallisto [4], or STAR [8]. Examples of ancillary tools include the SRAToolkit [24] for data retrieval from the NCBI SRA, Trimmomatic [3] for contaminant and quality trimming (HISAT2/STAR workflows), SAMtools [19] for storing alignments, Stringtie [27]) for read counting (HISAT2/STAR workflow) and quality analysis reports such as FastQC [2] and MultiQC [9].

Several automated RNA-seq workflows have been created to ease the burden of managing the steps of RNA-seq processing. These include Pipelines in Genomics (PiGx) [40], Visualization Pipeline for RNA sequencing analysis (VIPER) [5], handy parameter-free pipeline for RNA-Seq analysis (hppRNA) [36], Closha [15], the Transparent Reproducible and Automated PipeLINE (TRAPLINE) [39] and the nf-core/rnaseq workflow (Phil [28].

A popular advancement in workflow construction is the use of framework software to construct and then manage execution of the workflow. Popular examples include Galaxy [1], Kepler [21], Nextflow [7] and Snakemake [16]. Workflow managers simplify workflow construction and ensure automation with reproducible results, and often provide automatic execution on a variety of computing platforms. For example, Nextflow can manage execution of workflows on desktop computers or HPC systems such as Grid Engine [12], Portable Batch System (PBS) [11], HTCondor [33], SLURM [13], Kubernetes [35], popular commercial cloud platforms, and others. Nextflow also uses containers, such as Docker [22] and Singularity [17] to encapsulate dependent software for the workflow, eliminating the need for installation of software and managing interdependencies. Containerization ensures that software versions are consistent, ensuring reproducible results even when the workflow is executed on different computing platforms. One benefit of workflow frameworks is when larger datasets are used, researchers are not required to rewrite a workflow when moving to a different computing platform. Additionally, workflows built with containerized software can run simultaneously on multiple platforms.

To assist bioinformaticians in the development of portable, standards-based reproducible workflows, the nf-core framework [10] was developed which provides workflow construction standards, peer-review and best-practice recommendations for workflows constructed using Nextflow. The nf-core provides an interactive community of developers accessible via online communication tools to assist others in development of workflows. It consists of many released workflows and a variety of others that are under construction. These include the RNA-seq workflow: nf-core/rnaseq.

Here we introduce an RNA-seq workflow named GEMmaker. Despite the existence of other workflows, it grew from the need to process 26,055 SRA runs from 17,018 SRA experiments. Unfortunately, the nf-core/rnaseq workflow was not able to scale to this large dataset as it would exhaust available storage. When thousands of RNA-seq samples are used, intermediate files can exceed available compute storage as is the case of the HISAT2 tool which can quickly consume terabytes of storage when hundreds or thousands of samples require processing. Other gene quantification tools such as Salmon [26] and kallisto [4] require less data storage but can also exhaust storage depending on the number of samples.

The inability to scale without overrunning user data storage is a limitation of Nextflow rather than the nf-core/rnaseq workflow, which could overrun user storage—especially for large datasets. There are two key factors inhibiting scaling. First, Nextflow does not currently support cleanup of intermediate files. Second, Nextflow tends to execute all instances of the same step (e.g., downloading of SRAs from NCBI) before moving to the next step (e.g., quantification with kallisto) compounding the challenge of cleanup of intermediate files since cleanup cannot occur until later steps are completed.

Until the time that Nextflow supports a file cleanup strategy, a solution is needed to support RNA-seq workflows that need to scale without overrunning storage. Ideally, the solution would be to contribute code to the nf-core/rnaseq workflow to support file cleanup, but the nf-core standards require that workflows only support native Nextflow functionality. GEMmaker, therefore, exists to provide a workflow that supports massive scaling of RNA-seq processing when storage is limited. GEMmaker v2.1 is fully nf-core compatible and can be used in the same manner as any nf-core workflow. It provides much of the functionality of the nf-core/rnaseq workflow as well as the portability and reproducibility benefits inherit with Nextflow and nf-core workflows. GEMmaker is not better than other workflows in terms of accuracy of results or improved computational time, so we do not compare it to other workflows. Rather, it is meant to process increasingly large datasets without overrunning storage using the same steps that are common in other RNA-seq workflows. The following describes the implementation of GEMmaker and provides storage performance results.

## Implementation

GEMmaker uses Nextflow and is a combination of Groovy scripts for interfacing with Nextflow, Python scripts for wrangling intermediate data, and Bash scripts for execution of each software tool in the workflow. Nextflow was selected as the framework because it is widely used, is well supported, has a robust community of workflow creators in the life sciences, supports multiple computing platforms and supports containerization systems such as Docker and Singularity. Nextflow allows for execution of workflows from a command-line interface, which is common with most HPC platforms. These attributes make GEMmaker relatively easy to use. The following is an example command-line for execution of GEMmaker on a local machine using Singularity (for containerization), quantification using Salmon, and a file containing a list of SRA run IDs for *Arabidopsis thaliana* Illumina datasets:
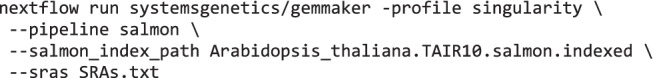


GEMmaker adopts the nf-core recommendations and standards to provide consistency in functionality with other popular nf-core workflows.

**Fig. 1:**
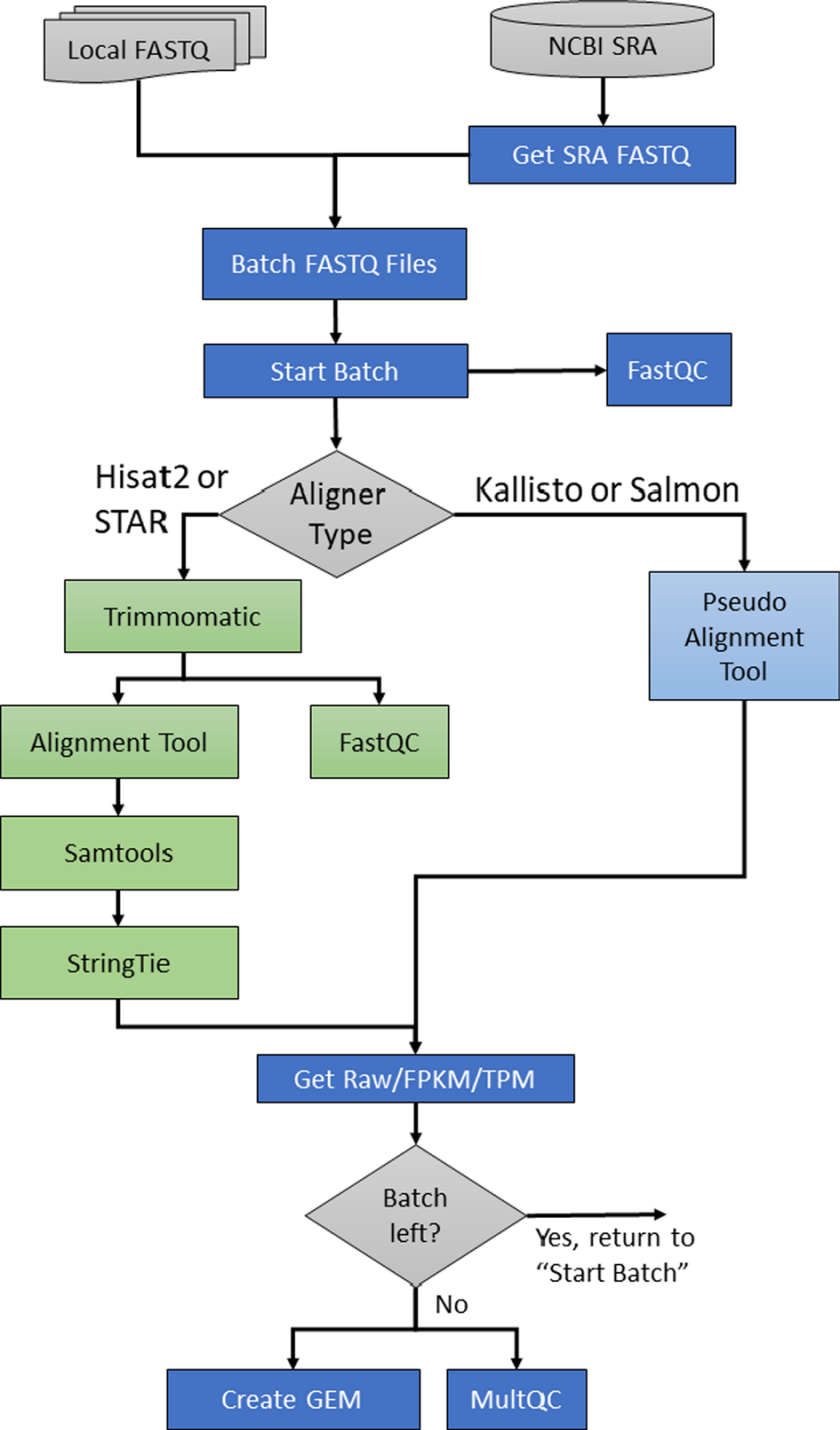
GEMmaker workflow diagram. GEMmaker supports the inclusion of both local and remote RNA-seq data files and offers four different alignment tools for gene expression quantification: Hisat2, STAR, Kallisto, and Salmon

GEMmaker uses a variety of software tools for gene expression-level quantification and quality control that can be selected by the user. These software are listed in Table 1 and the step-by-step flow of the workflow using these tools is shown in Fig. 1. There are four primary paths for gene expression quantification within GEMmaker: STAR, HISAT2, Salmon and kallisto. The STAR and HISAT2 paths include read trimming via Trimmomatic, SAMtools for storing alignments and Stringtie for quantification. Salmon and kallisto do not require those steps. All paths provide a MultiQC report to help end-users explore the quality of results from the workflow.

**Table 1:** Containerized software tools used in release v2.0 of GEMmaker

Tool	Version	Notes
nf-core/base	1.13.3	The base operating system for all nf-core compatible workflows
Python3	3.9.2	Used by a variety of custom data wrangling tools
Aspera	3.8.1	Downloads SRA files from NCBI SRA using provided run IDs
SRAToolkit	2.10.0	Downloads SRA files from NCBI using provided SRA Run IDs
FastQC	0.11.9	Generates read quality statistics for FASTQ files
Trimmomatic	0.39	Removes low-quality bases and removes adapter sequences
STAR	2.7.9a	Aligns cleaned reads to the reference
HISAT2	2.2.0	Aligns cleaned reads to the reference
Salmon	1.5.2	Performs quasi-alignment of reads and quantities
kallisto	0.46.2	Performs pseudo-alignment of reads and quantities
SAMTools	1.14	Used for indexing and sorting of BAM files created by HISAT2
StringTie	2.1.7	Performs gene expression quantification
MultiQC	1.11	Generate a full summary report for the entire workflow

As mentioned previously, GEMmaker is designed to scale. It can scale to process increasingly larger experiments (or large numbers of samples from public repositories) that can include hundreds to thousands of RNA-seq samples without intermediate files overrunning available compute storage. It supports execution on a large variety of computational platforms such that researchers can take full advantage of the compute facilities available to them including local desktop workstations, institutional clusters, national-funded resources such as XSEDE [34], the Pacific Research Platform [31], and commercial clouds.

To ensure storage requirements are not exceeded, GEMmaker moves input FASTQ files between three folders: “stage”, “processing” and “done”. Initially all samples are placed in the “stage” folder and GEMmaker will move into the “processing” folder as many samples as there are CPUs available. The user sets the number of CPUs that the workflow can use with the *–max_cpus* argument. On a compute cluster, this could be tens to hundreds. Nextflow is then instructed to automatically begin processing any samples that appear in the “processing” folder. As usual, Nextflow will process samples in parallel, using all CPUs, by first executing the first step for all samples, then the second for all samples, and so forth. However, because GEMmaker limits the number of samples to the number of CPUs, when a sample completes a step, it will move to the next step because Nextflow does not see any samples waiting. When a sample fully completes all steps, GEMmaker will then move the sample from the “processing” folder into the “done” folder and will move one sample from the “stage” folder into the “processing” folder. Nextflow sees this new sample in the “processing” folder and immediately begins processing that sample through each step. There is no lag between the time one sample finishes, and another begins and Nextflow should keep all CPUs consistently busy processing samples in parallel. As the workflow progresses for each sample, GEMmaker will cleanup unwanted intermediate files. This ensures space is cleaned before more samples begin processing. If the user specifies a *–max_cpu* size that does not exceed the resources of the computational platform, then GEMmaker can successfully process hundreds to thousands of samples.

While GEMmaker, by default, cleans all intermediate files, there are arguments that can be provided, as described in the online documentation, to control which intermediate files are removed. Users can keep downloaded SRA and FASTQ files, trimmed FASTQ files, SAM and BAM alignment files, and kallisto and Salmon pseudoalignment files. If any of these files are needed for downstream analyses they can be retained.

The speed at which the samples are processed depends on the number of processors and available memory of the compute nodes. Users with limited CPUs or RAM may need more time to process all samples. If users set the *–max_cpus* setting higher than storage will support, then GEMmaker may not be able to cleanup intermediate files before overrunning storage. It is difficult to recommend a value which maximizes the trade-off between the number of CPUs and storage requirements because RNA-seq samples and genomic reference sequences can be dramatically different in size, resulting in different sized intermediate files. However, using averaged values from the sample data reported here, we provide a rough recommendation that users have about 30 times the storage of an average sample size, times the number of CPUs when using HISAT2. For an average sample size of 2.5GBs this would require 75 GB per CPU. For kallisto and Salmon we recommend 7 times the storage of an average sample per CPU (17GBs).

To ensure portability between HPC systems, GEMmaker makes use of containerized software. This alleviate the burden of installing the same software versions on every computational system on which it is run. All GEMmaker dependent software are provided in the GEMmaker docker image and their versions are listed in Table 1. GEMmaker retrieves this Docker image from Docker Hub the first time it is run—users need not install any software other than Nextflow and a containerization software (Singularity or Docker). Thus, a GEMmaker workflow can be performed on any computational system and results will be reproducible and consistent.

## Results

We tested GEMmaker on WSU's Kamiak cluster which uses the SLURM scheduler [13], Clemson University's Palmetto cluster which uses the PBS scheduler [11], the Rodeo Kubernetes cluster at the Texas Advanced Computing Center (TACC) which contains homogenous set of compute nodes, and the Pacific Research Platform’s Nautilus cluster which contains a heterogenous set of compute nodes. In all platforms GEMmaker successfully completed. Because data storage usage is of most importance, GEMmaker was tested using two different datasets: a publicly available 475-sample *Oryza sativa* (rice) RNA-seq dataset (NCBI SRA accession PRJNA301554) [38], and the *Arabidopsis thaliana* 26,055-runs from NCBI.

**Fig. 2:**
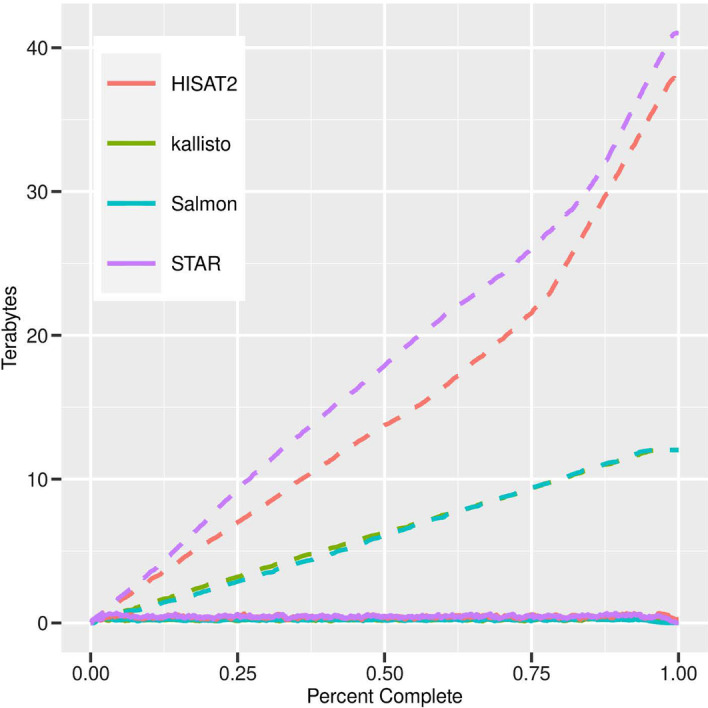
Storage usage comparison. Storage sizes for processing the 475-sample time-series rice dataset is shown. Dashed lines indicate tests in which GEMmaker was configured to not cleanup of intermediate files between batches, while solid lines indicate that a cleanup was performed

The 475 rice dataset consists of samples from two subspecies of rice, subdivided into 4 genotypes, grown in a hydroponic environment that underwent treatments of heat stress, drought stress and control. Measurements were taken every 15 min for several hours with 2 replicates. We selected this dataset to demonstrate execution of a large single experiment on a typical stand-alone workstation that researchers may have available to them. The Arabidopsis 26,055 dataset was selected using all Illumina RNA-seq datasets available at the time the list was collected. An SRA experiment can contain multiple runs which resulted in 17,018 SRA experiments. This included both paired and non-paired RNA-seq runs for *Arabidopsis thaliana* sequenced using the Illumina platform. The list of SRA run IDs is provided as Additional file 1: Data 1. We selected all RNA-seq data to test massive scale processing on a typical institutional HPC cluster. The 475 rice dataset was tested on Washington State University’s HPC cluster, Kamiak. To simulate execution on a stand-alone workstation, the job was limited to 16 CPUs and 6 GB of RAM (a reasonable set of resources for a performant workstation). The compute node contained Intel(R) Xeon(R) Gold 6138 CPU @ 2.00 GHz processors, had 256 GB of RAM (although, only 6 GB were requested) with access to 650 TB of network attached storage to allow for as much expansion of storage as needed (although, this large storge size is not required as shown in Fig. 2). GEMmaker was executed twice for each quantification tool (STAR, HISAT2, kallisto and Salmon) once with cleanup of intermediate files turned on and again turned off. Because the primary performance metric of concern is storage usage, a monitoring script tracked the storage space consumed. Results of the test are found in Fig. 2.

With the option to clean intermediate files enabled, all the quantification tools consumed less than 1 Tb of storage. At maximum, HISAT2 consumed 680 GB, kallisto 322 GB, Salmon 342 GB, and STAR 701 GB. When intermediate files were not cleaned, both Salmon and kallisto consumed approximately 12 TB of storage, HISAT2 38 TB and Star 41 TB. Salmon and kallisto took less time (~ 3 days) than STAR (4 days), or HISAT2 (~ 5.5 days) to run. Compute time is strongly dependent on each computer’s hardware and the queue size. Therefore, this test could have run quicker if the number of CPUs were increased. The range of storage space (between 322 and 680 GB) required to execute GEMmaker on this set of 475 samples, with intermediate file cleaning enabled, is commonly available on stand-alone workstations.

To demonstrate processing of tens of thousands of RNA-seq datasets, the 26 K SRA runs were processed on WSU’s Kamiak HPC cluster with a *–max_cpus* setting of 120 (i.e., 120 currently running jobs in parallel). We used the kallisto pipeline, and GEMmaker completed processing the 26 K runs over 28 days. We designed GEMmaker so that if a dataset is corrupted, or if information was incorrectly entered into NCBI that it would report these and then continue with other samples. This reduces downtime and allows the user to look at these files manually. GEMmaker reported that of the 26 K runs, 19 SRA files had no metadata available via NCBI web services and could not be retrieved; 179 had missing download URLs; 3 samples were corrupted after download; and 1 failed to download due to a network timeout. Just as with the rice data, GEMmaker was instructed to clean intermediate files (SRA files, FASTQ files, kallisto index files, etc.) and keep only raw and TPM count files, but actual storage usage was not measured during runtime. The results folder consumed 48 GB of storage.

## Limitations

Despite the advantages that GEMmaker affords, it has limitations. First, we could not include every quantification tool made to date; users who need other tools are encouraged to request features on the GEMmaker GitHub issue queue. Second, if GEMmaker is preempted before it completes, as was the case with the 26 K Arabidopsis dataset, then there may be working directories that do not get cleaned. Because GEMmaker is a Nextflow workflow, it can resume execution where it left off. However, Nextflow creates new working directories for each step of the workflow for each sample and when it is resumed it creates new working folders—the folders with failed steps remain. When a sample completes a step, then GEMmaker can clean up the working directories that were successful but there is not a mechanism in Nextflow to know about the directories with failed results so that they can be cleaned. As a result, if a high *–max_cpus* is used (e.g., 120) and Nextflow is preempted this may result in higher storage usage from directories with failed jobs. Third, related to usability, GEMmaker does not have a graphical user interface (GUI). Users familiar with the UNIX command line will not see this as an issue, but those who have limited experience may find this difficult. Finally, GEMmaker was not designed for data security. Users with sensitive data will need to coordinate with data security experts to ensure processing is executed in a secure facility.

## Conclusion

GEMmaker addresses issues of scale for processing massive RNA-seq experiments with hundreds to thousands of samples (although it can be used for small datasets as well). While automated RNA-seq workflows already exist, GEMmaker is unique in that it does not overrun data storage facilities yet provides similar functionality to that of gold-standard RNA-seq workflows. GEMmaker allows researchers to take advantage of existing smaller computing infrastructure which can be beneficial if there is limited access to larger facilities. GEMmaker returns count data in various formats (e.g., raw and normalized) so that results can be used in downstream transcriptome analyses such as differential gene expression, regulatory network construction and gene co-expression analysis.

### Availability and requirements

Project name: GEMmaker

Project home page: https://github.com/SystemsGenetics/GEMmaker

Operating systems(s): Platform independent

Programming language: Nextflow Groovy, Python and bash

Other requirements: Nextflow and Java. Docker or singularity are optional but suggested

Any restrictions to use by non-academics: GPL v2.0 license.

## Supplementary Information

**Additional file 1:** NCBI SRR IDs.

## Data Availability

GEMmaker is freely available at https://github.com/SystemsGenetics/GEMmaker and is accompanied by full step-by-step documentation online at https://gemmaker.readthedocs.io/en/latest including instructions for genome preparation. GEMmaker Docker images are available on Docker Hub at https://hub.docker.com/u/gemmaker.

## Abbreviations

HPC: High performance computing
RNA-seq: RNA sequencing
SRA: Sequence read archive
